# Exposure of Breast and Lung Cancer Cells to a Novel Estrone Analog Prior to Radiation Enhances Bcl-2-Mediated Cell Death

**DOI:** 10.3390/ijms19102887

**Published:** 2018-09-23

**Authors:** Elsie M. Nolte, Anna M. Joubert, Roy Lakier, Ado van Rensburg, Anne E. Mercier

**Affiliations:** 1 Department of Physiology, School of Medicine, Faculty of Health Sciences, University of Pretoria, Pretoria 0002, South Africa; annie.joubert@up.ac.za (A.M.J.); joji.mercier@up.ac.za (A.E.M.); 2 Department of Radiation Oncology, Steve Biko Academic Hospital, Faculty of Health Sciences, B6L2007 Oncology Block, University of Pretoria, Pretoria 0002, South Africa; roy.lakier@up.ac.za (R.L.); Ado@cmnuclear.co.za (A.v.R.)

**Keywords:** cancer, radiation therapy, 2-methoxyestradiol, ESE-15-ol, radiosensitization, apoptosis, Bcl-2, reactive oxygen species, clonogenic studies

## Abstract

Following exposure of cells to gamma-radiation, a cascade of intracellular consequences may be observed in a semitemporal manner. This includes deoxyribonucleic acid (DNA) damage and reactive oxygen species (ROS) accumulation initially, with consequent signaling for DNA repair and facilitative regulation of the cell cycle. Failure to rectify the damage or ROS levels leads to induction of senescence or apoptosis. 2-Ethyl-3-*O*-sulfamoyl-estra-1,3,5(10),15-tetraen-17-ol (ESE-15-ol), a 2-methoxyestradiole analog designed in silico for superior pharmacokinetics, was investigated for its potential to enhance apoptotic signaling and decrease the long-term survival of cells exposed to radiation. Sequential early intracellular effects within radiation-treated MCF-7 breast- and A549 lung cancer cells pre-exposed to low-dose ESE-15-ol were investigated using various flow cytometric protocols, spectrophotometry, and microscopy. Long-term cellular survival and proliferation was examined using clonogenic studies, which demonstrated a significant decrease in the presensitized cells. Combination-treated cells exhibited increased superoxide formation, and decreased Bcl-2 expression and -phosphorylation. Induction of apoptosis and elevation of the sub-G_1_ phase was evident in the pre-exposed MCF-7 cells, although only minimally in the A549 cells at 48-h. These results indicate that low-dose ESE-15-ol may increase tumor response to radiation. Future studies will investigate the effect of ESE-15-ol pre-exposure on radiation-induced DNA damage and repair mechanisms.

## 1. Introduction

Cancer treatment regimens, particularly for small cell lung cancer and both *in situ-* and metastasized breast carcinomas, may involve a combination of chemotherapeutics and radiation. Chemical agents which alter nucleotide metabolism, such as fluoropyrimidines, have proven their ability to sensitize gastrointestinal neoplasias both in vitro and in vivo [1]. These agents presensitize malignant cells by impairing the function of the S-phase checkpoints [2]. Additionally, gemcitabine may affect deoxyribonucleic acid (DNA) repair pathways, whereas cisplatin may work via multiple mechanisms including adduct formation [2]. Paclitaxel, a microtubule stabilizing agent, has also demonstrated the capacity to radiosensitize various human cell carcinomas [3]. Authors concluded that cells must be in a G_2_/M-phase block for maximal radiation effects in MCF-7 breast carcinoma cells, whereas A549 lung cancer cells remained unsensitized irrespective of cell cycle phase [3]. Although phase I/II clinical trials using pulsed low-dose paclitaxel as a radiosensitization agent for thoracic malignancies showed promise [4], certain cell types such as human breast (MCF-7) and colon (HT-29) carcinomas failed to demonstrate a G_2_/M block as a result of the paclitaxel exposure [5]. Furthermore, paclitaxel presensitization was associated with a high occurrence of unwanted effects such as pneumonitis and esophagitis, postulated to be due to sensitization of the normal untransformed surrounding tissue to the radiation [4,6].

A metabolite of 17β-estradiol, 2-methoxyestradiol (2ME2), has the ability to inhibit proliferation of cancer cells [7]. 2ME2 has demonstrated cytotoxicity in approximately 55 different tumor cell lines in vitro [8]. Moreover, 2ME2 partially spares noncancerous cells in favor of active proliferating malignant cells [8]. 2ME2 induces apoptosis via both the intrinsic- and extrinsic pathways. But unlike classic spindle poisons such as the vinca alkaloids and paclitaxel, 2ME2 does not act as a substrate of the P-glycoprotein (PgP) pumps [9]. This makes the compound a potential candidate in the treatment of multidrug-resistant cancer types [4,5,10]. Several in vitro and in vivo mechanistic studies demonstrated that 2ME2 acts as a microtubule disruptor via drug-binding to the colchicine site [11]. This results in the formation of abnormal spindles, as well as mitotic accumulation [12]. 2ME2 exerts its anticancer effects independently of cellular estrogen receptors and displays no systemic hormonal effects [13,14].

As the G_2_/M phase of the cell cycle renders the cells most vulnerable to radiation, spindle poisons such as 2ME2 which induce this mitotic block may serve as a potential mechanism to confer radiosensitivity in a pretreatment strategy [15,16]. Casares et al. [17] evaluated the potential radiosensitization of prostate cancer models by 2ME2, as this cancer type not only shows sensitivity to 2ME2 monotherapy, but is also treated frequently with radiation. Authors determined that mitogen-activated protein kinase (MAPK) phosphorylation decreased in a dose-dependent manner when PC3 prostate cancer cells were treated with 2ME2 for 18-h [17]. Involvement of this signaling cascade in the radiosensitization mechanism was confirmed by selective inhibition of MAPK/extracellular signal regulated kinase kinase (MEK 1/2), an upstream effector of MAPK [18]. The decrease in MAPK phosphorylation correlated with decreased colony formation in the presensitized PC3 cells, together with decreased survival. Furthermore, in vivo orthotopic experiments on male nude mice inoculated subcutaneously with PC-3M-luc-C6 prostate cancer cells which were treated with 75 mg/kg 2ME2 (oral administration) for 4-h prior to 3 Gy radiation, displayed a synergistic decrease in the tumor growth with the two treatments [17].

2ME2 undergoes 17β-hydroxysteroid dehydrogenase-mediated metabolism and is thus rapidly metabolized, resulting in a low oral bioavailability. Consequently, Stander et al. [19] designed sulfamoylated 2ME2 analogs in silico to improve both the pharmacodynamic-, as well as the potential pharmacokinetic profile of the parent compound. The design aimed to improve the specificity and affinity of the molecular interaction at the microtubule colchicine site, thereby increasing the drug’s toxicity. Additionally, design aimed at enhancing carbonic anhydrase IX (CAIX) binding, an enzyme active within the acidic tumor micromilieu, thus potentially localizing the compounds to the tumor [11,20,21]. Addition of the sulfamate moiety at position 3 allows reversible binding to erythrocytic CAII, extending the half-life by bypassing the fist-pass liver metabolism [22,23]. These novel analogs displayed cytotoxicity at nanomolar concentrations in various cancer cell lines including a multiple drug resistant sarcoma cell line [9]. The analogs demonstrated microtubule disrupting effects and induced apoptosis via both the intrinsic- and extrinsic pathways [9,24]. One of these analogs, 2-ethyl-3-*O*-sulfamoyl-estra-1,3,5(10),15-tetraen-17-ol (ESE-15-ol), holds therapeutic promise as an anticancer drug, but it is currently not commercially available. The ability of ESE-15-ol to enhance cell death signaling in cancer cells in response to radiation treatment has not been previously investigated. Particularly, the goal to minimize the dose of both drug and radiation to curtail the side effect profiles while maximizing each modalities’ therapeutic effect would be ideal. Thus, the aim of this study was to determine whether low-dose ESE-15-ol would augment apoptotic signaling and decrease long-term survival of estrogen receptor-positive MCF-7 breast cancer cells, as well as A549 human lung carcinoma cells in response to radiation treatment.

## 2. Results

### 2.1. Cytotoxicity Studies

Dose-response curves indicated a half-maximal inhibitory growth concentration (GI_50_) of 0.333 ± 0.12 µM and 0.111 ± 0.01 µM in A549- and MCF-7 cell lines respectively, after exposure to ESE-15-ol for 24-h (Table 1). These values did not differ in the 48-h exposure.

### 2.2. Apoptosis Studies: Annexin V-FITC

The lowest dose of drug and radiation that individually decreased cell viability significantly was determined by a dose curve (48 h-exposure) and quantified by annexin V-propidium iodide staining via flow cytometry. Values obtained for ESE-15-ol exposure were 0.081 µM for A549 cells and 0.056 µM for MCF-7 cells (Appendix A). These concentrations were used subsequently to presensitize the cells for 24-h prior to 6 Gy radiation. Experiments were terminated 48-h after radiation. Cell viability was decreased to less than 80% after 48-h in both cell lines in response to 6 Gy radiation (Appendix B).

**Figure 1:**
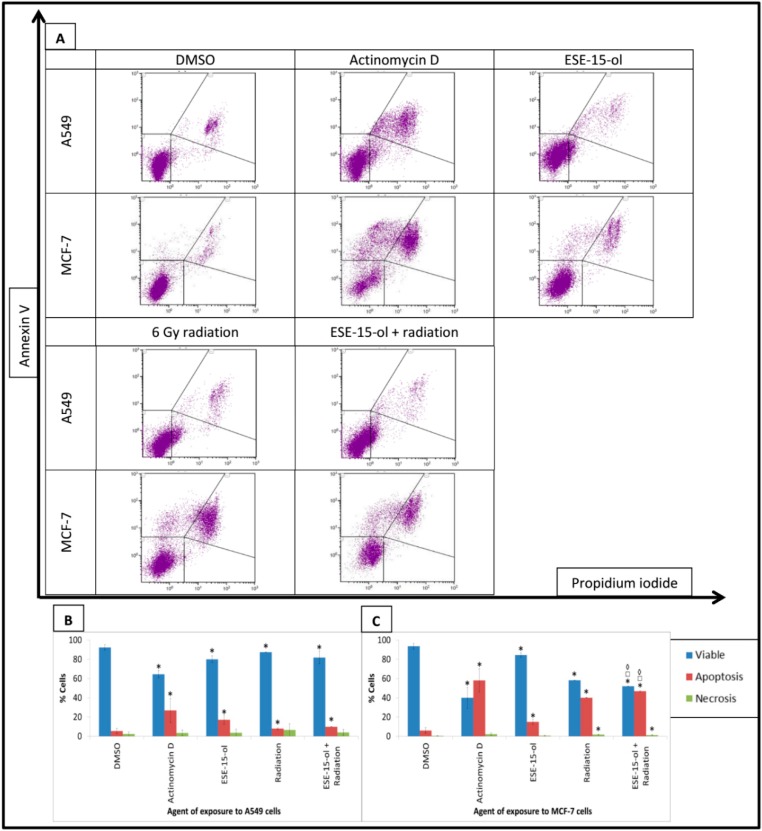
Annexin V-FITC detection in A549- and MCF-7 cells. Annexin V-FITC (FL1) measured apoptotic cells and was plotted against propidium iodide (FL3). (**A**) Dot plots of A549- and MCF-7 cells exposed to ESE-15-ol for 24-h, 6 Gy radiation, and the combination treatment condition. DMSO served as a vehicle control and actinomycin D as a positive method control for apoptosis. A549- and MCF-7 cell viability decreased while apoptosis increased in all treated samples, an effect more pronounced in presensitized MCF-7 cells. Graphical representations of A549 dot plots (**B**) and MCF-7 dot plots (**C**). Bars indicate average of three biological repeats, with standard deviation represented by T-bars. Statistical significance (*p*-value < 0.05) represented by * when compared to DMSO, □ when compared to ESE-15-ol treated samples, ◊ when compared to radiation treated samples.

Apoptosis studies were conducted on A549 and MCF-7 cells to determine whether a 24-h-pre-exposure to low-dose ESE-15-ol increased apoptotic cell death 48-h after 6 Gy radiation. Enhanced apoptotic effects of the combination treatment were not clearly evident at this time-point in the A549 cells, which showed a similar decrease in cell viability and increased in phosphatidylserine flip in all treated samples when compared to the DMSO vehicle control (24-h ESE-15-ol-exposure (viability 80.1 ± 3.55%; apoptosis 17.12 ± 4.68%); 6 Gy radiation (viability 87.37 ± 0.23%; apoptosis 7.9 ± 1%); and the combination treatment (viability 81.83 ± 6.63%; apoptosis 9.76 ± 0.26%)) (Figure 1). However, the effect of the treatments were more pronounced in the MCF-7 cells (ESE-15-ol for 24-h (viability 84.41 ± 2.25%; apoptosis 14.95 ± 2.62%); 6 Gy radiation (viability 58.19 ± 0.06%; apoptosis 40.13 ± 0.37%) and the combination treatment (viability 51.98 ± 0.28%; apoptosis 46.79 ± 0.67%)). Furthermore, there was a small but significant increase in necrosis in MCF-7 cells exposed to 6 Gy radiation (1.69 ± 0.43%) and the combination treatment condition (1.23 ± 0.4%) compared to the DMSO vehicle control (0.46 ± 0.09%). MCF-7 cells exposed to the combination treatment demonstrated a significant decrease in cell viability with a concurrent increase in the apoptotic fraction when compared to the individual exposures, a finding not demonstrable in the A549 cells at this time-point.

### 2.3. Cell Cycle Analysis

**Figure 2:**
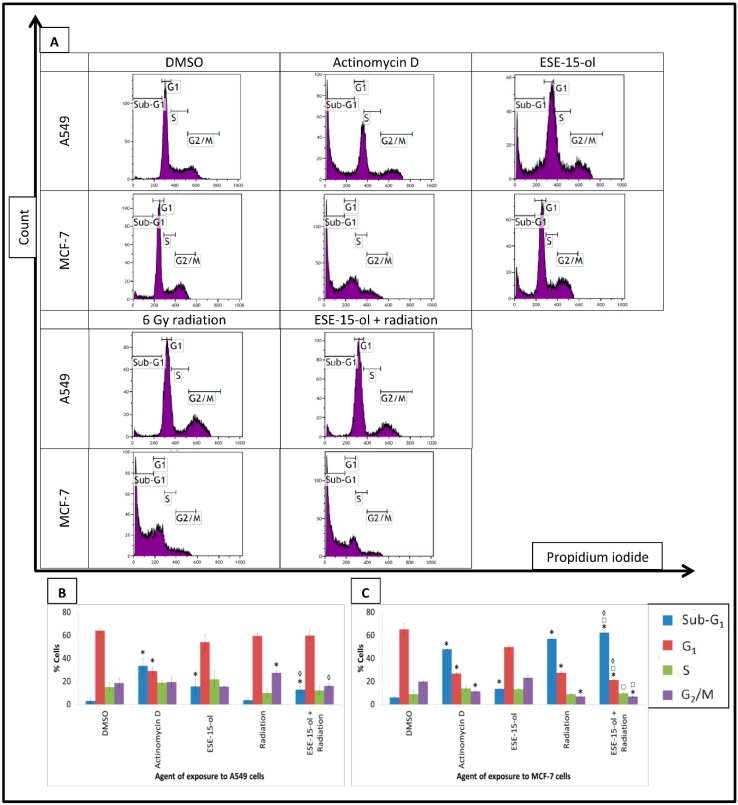
Cell cycle analysis of A549- and MCF-7 cells. Propidium iodide (FL3) was plotted against number of fluorescent events to analyze the cell cycle distributions. (**A**) Dot plots of A549 and MCF-7 cells exposed to ESE-15-ol for 24-h, 6 Gy radiation, and the combination treatment condition. DMSO served as a vehicle control and actinomycin D as a positive method control for apoptosis. Presensitized MCF-7 cells displayed a more prominent increase in sub-G_1_ and a decrease in G_1_ when compared to cells exposed to ESE-15-ol and 6 Gy radiation. Graphical representations of A549 dot plots (**B**) and MCF-7 dot plots (**C**). Bars indicate the average of three biological repeats, with standard deviation represented by T-bars. Statistical significance (*p*-value < 0.05) represented by * when compared to DMSO, □ when compared to ESE-15-ol treated samples, ◊ when compared to radiation treated samples.

Cell cycle analysis was done using propidium iodide quantification via flow cytometry. A549- and MCF-7 cells exposed to DMSO revealed that 64.01 ± 2.01% and 65.16 ± 5.38% of the cells were in the G_1_ phase, respectively (Figure 2). A549 cells treated with the combination therapy displayed similar responses to the individual treatments. The sub-G_1_ phase was significantly increased in A549 cells exposed to ESE-15-ol for 24-h (15.57 ± 6.76%) and the combination treatment condition (12.79 ± 2.76%) when compared to DMSO (2.94 ± 1.82%). Response to the combination therapy was more evident in the MCF-7 cells, with 13.58 ± 0.01% cells in the sub-G_1_ phase when exposed to ESE-15-ol, 56.83 ± 0.26% after 6 Gy radiation, and 62.23 ± 1.17% with the combination treatment (DMSO 6.14 ± 0.64%), with a concurrent significant decrease in the G_1_ phase. A549 cells exposed to 6 Gy radiation (27.36 ± 1.75%) demonstrated a significant decrease in G_2_/M. When compare to the 6 Gy radiation control, presensitized A549- and MCF-7 cells demonstrated a significant increase in sub-G_1_ (3.69 ± 1.4% and 56.83 ± 0.26%, respectively). This population of cell may not only indicate apoptotic cells, but may also represent cells with increased DNA fragmentation and micronuclei formation [25]. At this time point, the MCF-7 cells demonstrated a further progression of apoptosis in the presensitized cells as the combination treatment increased the sub-G_1_ population significantly more than the drug and radiation controls, with a concomitant decrease in G_1_.

### 2.4. Polarization-Optical Transmitted Light Differential Interference Contrast Microscopy (PlasDIC)

In order to determine the temporal morphological consequences of the treatment conditions, PlasDIC images of A549- and MCF-7 cells were taken at different time intervals.

**Figure 3:**
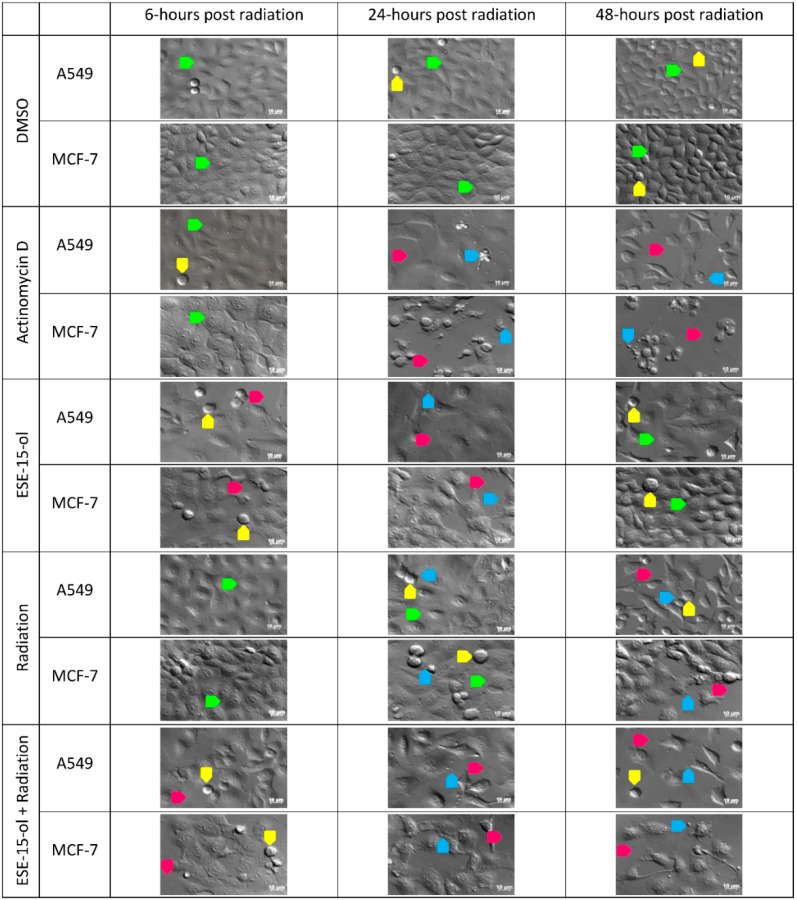
PlasDIC images of A549- and MCF-7 cells. A549- and MCF-7 cells were exposed to control and treatment conditions over a 48-h time period. Images illustrate cells in interphase (green arrow), metaphase (yellow arrow), apoptotic bodies (blue arrow), and decreased cell density (pink arrow). Scale bars represent 10 µm.

A549- and MCF-7 cells exposed to DMSO continued to grow in a logarithmic fashion as seen in an increase in cell density from 6-h to 48-h after radiation (Figure 3). A549- and MCF-7 cells exposed to ESE-15-ol for 24-h (before washing it off and replacing with growth medium for 48-h) revealed some rounded cells. Similar to the positive control, cell density was decreased in the ESE-15-ol treated samples, and apoptotic bodies were observed. Cell density of cells exposed to ESE-15-ol for 24-h increased over the 48-h period after removal of the compound, demonstrating the reversible action of this compound [24]. An increased number of cells present in metaphase were observed in A549- and MCF-7 cells exposed to 6 Gy radiation, and apoptotic body formation was observed from 24-h. A549- and MCF-7 cells exposed to ESE-15-ol prior to radiation revealed a decrease in cell density over the 48-h time frame. Apoptotic body formation was observed at 24-h as well as 48-h after radiation, more so in the MCF-7 cells (Figure 3). Noted was the presence of enlarged, flattened cells possibly indicative of senescence.

### 2.5. Transmission Electron Microscopy

Transmission electron microscopy was used to visualize ultrastructural changes caused by ESE-15-ol, 6 Gy radiation, as well as cells exposed to ESE-15-ol 24-h prior to radiation. 

**Figure 4:**
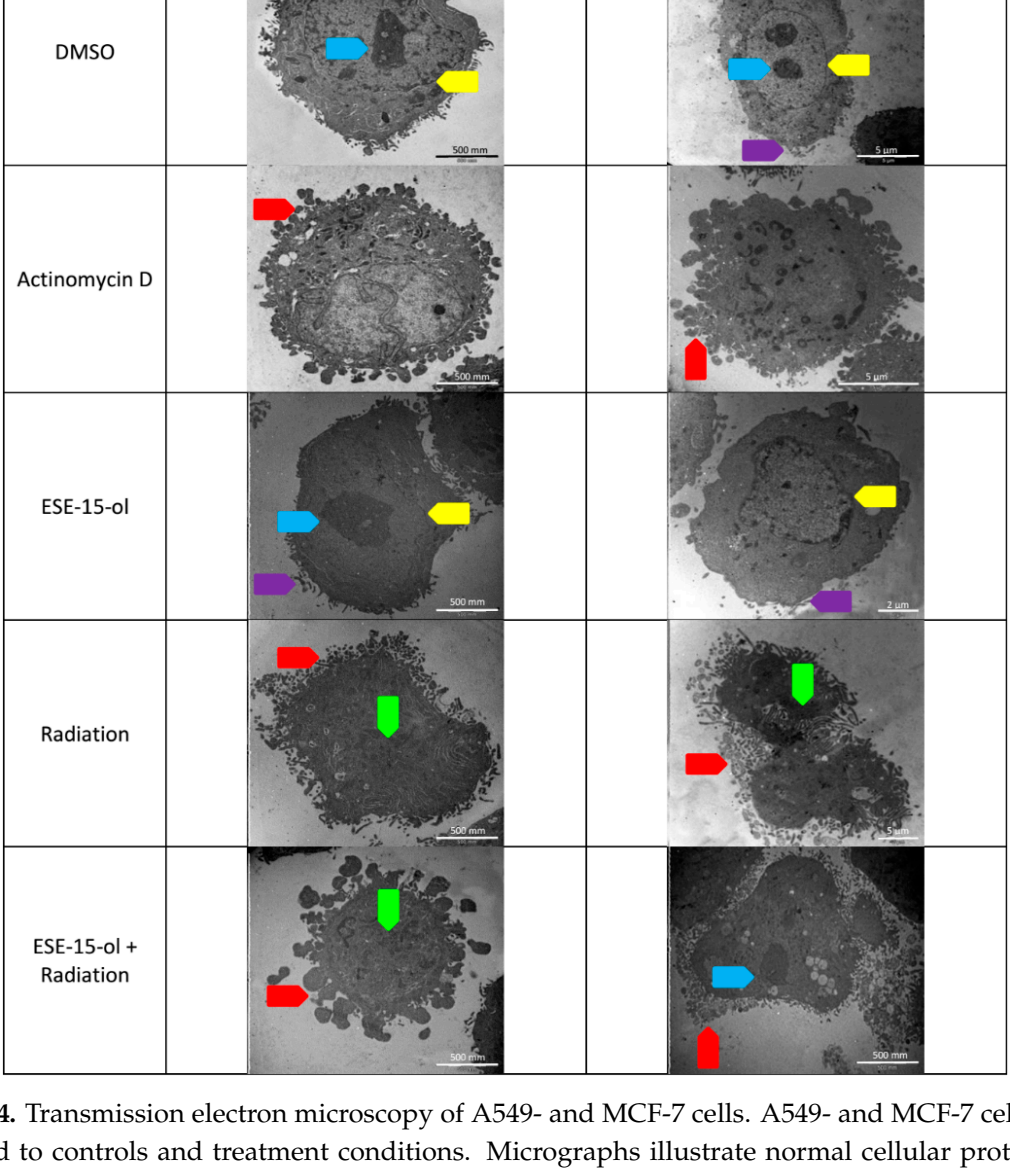
Transmission electron microscopy of A549- and MCF-7 cells. A549- and MCF-7 cells were exposed to controls and treatment conditions. Micrographs illustrate normal cellular protrusions (purple arrow), nucleoli (blue arrow), nuclear membrane (yellow arrow), hypercondensed chromatin (green arrow), and increased cellular protrusions (red arrow).

Transmission electron micrographs of A549- and MCF-7 cells revealed a smooth cell membrane with normal cellular protrusions in cells exposed to DMSO (Figure 4). A549- and MCF-7 cells exposed to ESE-15-ol for 24-h, after which the drug was removed for 48-h, presented with a smooth cellular membrane similar to the negative control. A549- and MCF-7 cells radiated with 6 Gy presented with similar morphology as the positive control, which included hypercondensed chromatin and increased cellular protrusions. A549- and MCF-7 cells presensitized by ESE-15-ol prior to radiation revealed hypercondensed chromatin and prominent cellular protrusions (Figure 4).

### 2.6. Clonogenic Studies

Clonogenic studies were used to determine the cells’ ability to form colonies after different treatment conditions [26]. Data was adjusted according to initial number of cells seeded per treatment condition. Colony forming abilities were analyzed according the percentage of area covered by the colonies, as well as intensity which indicated the density of the colonies, thereby assessing the ability of the cells to survive, recover and proliferate after the exposures. 

**Figure 5:**
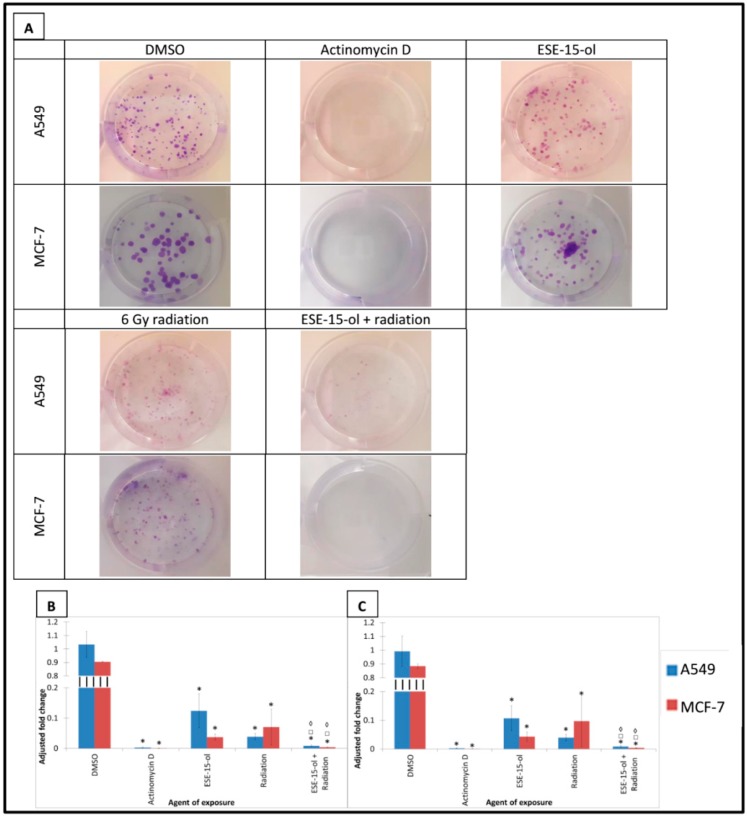
Clonogenic studies of treated A549- and MCF-7 cells. (**A**) Colony formation of A549- and MCF-7 cells exposed to ESE-15-ol for 24-h, 6 Gy radiation, and the combination treatment condition. DMSO served as a vehicle control and actinomycin D as a positive control for apoptosis. Colony formation decreased in all treatment conditions. (**B**) Graphical representations of area percentage covered by A549- and MCF-7 cells. (**C**) Graphical representation of intensity of A549- and MCF-7 colonies. Bars indicate the average of three biological repeats, with standard deviation represented by T-bars. Statistical significance (*p*-value < 0.05) represented by * when compared to DMSO, □ when compared to ESE-15-ol treated samples, ◊ when compared to the radiation treated samples.

The percentage area covered by colonies formed in A549- and MCF-7 cells exposed to the ESE-15-ol control decreased by 88 ± 0.056% and 96 ± 0.01%, while the intensity decreased by 89 ± 0.043% and 96 ± 0.016%, respectively (Figure 5). A549 cells radiated with 6 Gy displayed a decreased colony area coverage and intensity by 96 ± 0.011%. Irradiated MCF-7 cells displayed a decrease in area by 97 ± 0.06% and intensity by 90 ± 0.089%. A549 cells exposed to ESE-15-ol 24-h prior to radiation decreased their colony area and intensity by 99.9 ± 0.003% compared to the negative control. Similar to the positive control, no colony formation was observed in MCF-7 cells exposed to ESE-15-ol 24-h prior to radiation (Figure 5). Presensitized A549 cells significantly decreased in area percentage compared to ESE-15-ol treated samples (92 ± 0.06% decrease) and radiation treated samples (76 ± 0.09% decrease). Intensity of presensitized A549 colonies significantly decreased compared to ESE-15-ol and radiation treatment conditions by 91 ± 0.06% and 78 ± 0.09% respectively (Figure 5).

### 2.7. Superoxide Detection

**Figure 6:**
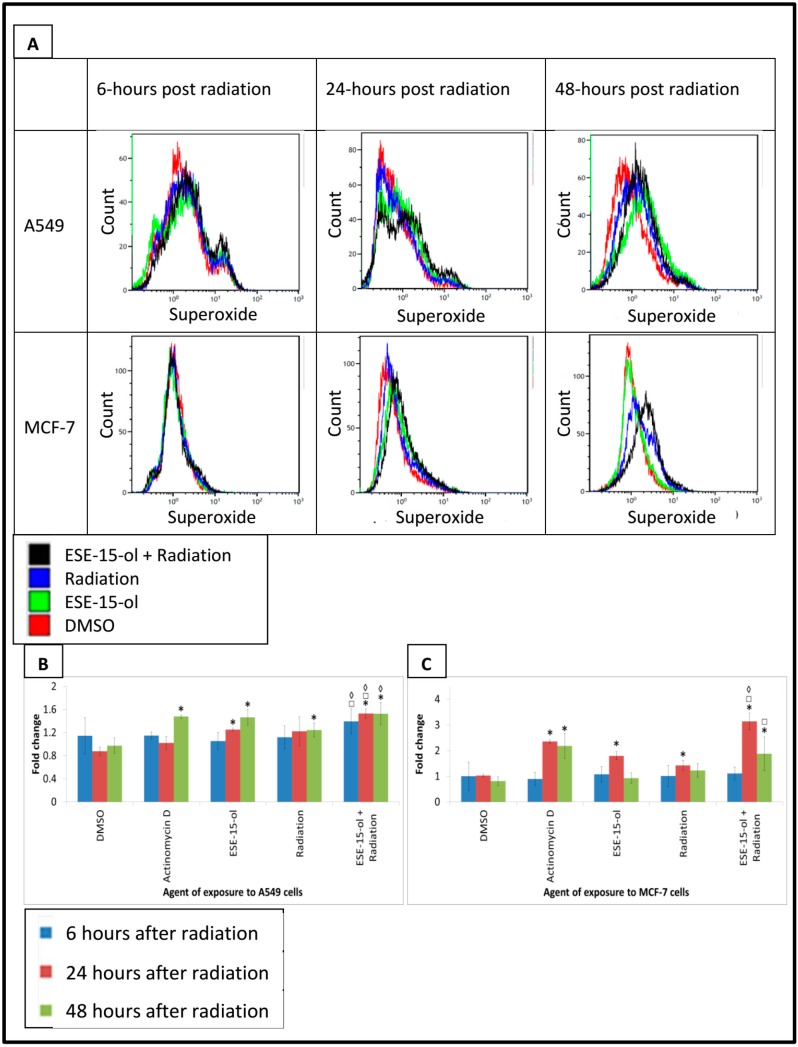
Superoxide detection in A549- and MCF-7 cells exposed to controls and treatment conditions. (**A**) Overlay histograms of A549- and MCF-7 cells exposed to DMSO, ESE-15-ol for 24-h, 6 Gy radiation, and the combination treatment condition. Histograms were obtained by plotting hydroethidine emission (FL3) against cell count. Superoxide levels were measured 6-h, 24-h, and 48-h after radiation. Superoxide levels were significantly increased at 24-h and 48-h in A549- and MCF-7 cells exposed to the different treatment conditions. (**B**) Graphical representations of superoxide detection in A549 cells. (**C**) Graphical representation of superoxide detection in MCF-7 cells. Bars indicate the average of three biological repeats, with standard deviations represented by T-bars. Statistical significance (*p*-value < 0.05) represented by * when compared to DMSO, □ when compared to ESE-15-ol treated samples, ◊ when compared to radiation only treated sample.

Superoxide was quantified by means of flow cytometry to evaluate reactive oxygen species generation [27] at 6-h, 24-h and 48-h after radiation. Superoxide was significantly increased at 24-h in MCF-7 cells exposed to ESE-15-ol (1.8 ± 0.16 fold) and radiation (1.4 ± 0.2 fold) alone (Figure 6). Presensitized A549- and MCF-7 cells also displayed an increase of 1.3 ± 0.02- and 3.1 ± 0.32 fold in superoxide radicles at the same time point. A549 cells exposed to ESE-15-ol (1.5 ± 0.13 fold) and radiation (1.2 ± 0.12 fold) demonstrated a statistically significant increase in superoxide levels at 48-h. Presensitized A549- and MCF-7 cells displayed an increase of 1.5 ± 0.19- and 1.9 ± 0.66 fold at 48-h, respectively. When compared to the drug-only control, presensitized A549 cells display significantly increased superoxide levels at 6-h (1.3 ± 0.02 fold) and 24-h (1.2 ± 0.1 fold). Presensitized MCF-7 cells displayed significantly higher levels of superoxide at 24-h (1.7 ± 0.03 fold) and 48-h (2 ± 0.37 fold) when compared to cells exposed to the drug alone. Superoxide detection was significantly increased in presensitized MCF-7 cells at 24-h (2.1 ± 0.15 fold) and presensitized A549 cells at 6-h (1.3 ± 0.05 fold), 24-h (1.4 ± 0.08 fold), and 48-h (1.3 ± 0.1 fold) compared the 6 Gy radiation control.

### 2.8. B Cell Lymphoma 2 (Bcl-2) Activation

**Figure 7:**
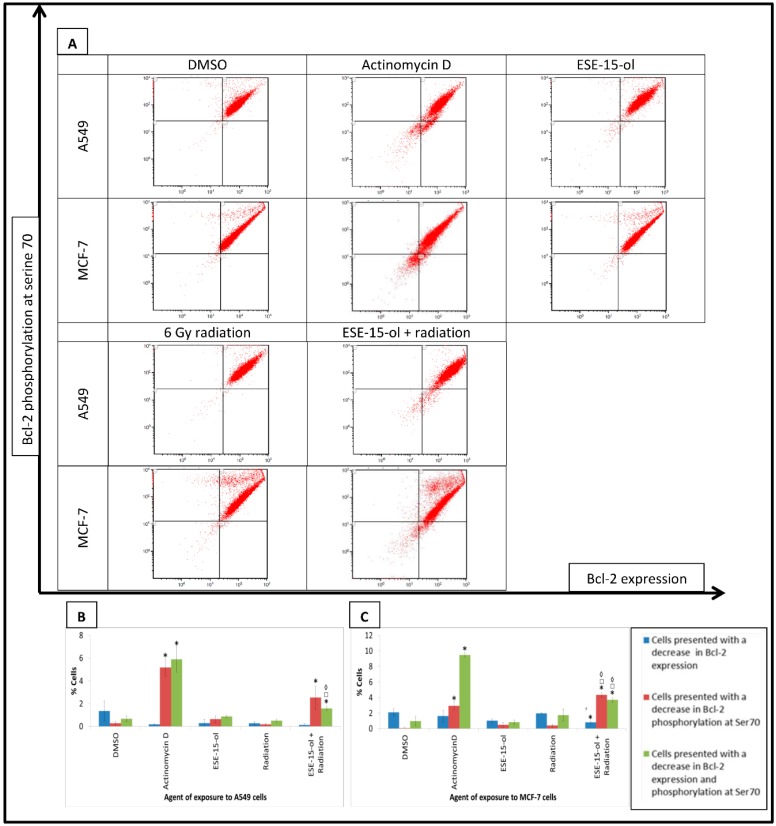
Flow cytometric analysis of Bcl-2 expression and -phosphorylation measured in exposed A549- and MCF-7 cells. (**A**) Fluorescence of FL1 (Bcl-2 expression) was plotted against FL3 (pBcl-2, Ser70). Dot plots of A549- and MCF-7 cells exposed to ESE-15-ol for 24-h, 6 Gy radiation and the combination treatment condition. Treated A549- and MCF-7 cells presenting with a decreased Bcl-2 expression and -phosphorylation were significantly increased when compared to DMSO. Presensitized A549- and MCF-7 cells displayed an increased number of cells with decreased Bcl-2 expression and -phosphorylation when compared to individual treatment conditions as shown in the graphical representations of A549 dot plots (**B**) and MCF-7 dot plots (**C**). Bars indicate average of three biological repeats, with standard deviation represented by T-bars. Statistical significance (*p*-value < 0.05) represented by * when compared to DMSO, □ when compared to ESE-15-ol treated samples, ◊ when compared to radiation treated samples.

B cell lymphoma 2 (Bcl-2) expression and -phosphorylation at serine 70 were quantified by means of flow cytometry in A549- and MCF-7 cells exposed to relevant treatment conditions and controls. A549- and MCF-7 cells exposed to ESE-15-ol for 24-h prior to radiation that revealed an increase in the proportion of cells that presented with a decreased phosphorylation of Bcl-2 (2.55 ± 1.02% and 4.35 ± 0.28%, respectively) compared to the vehicle controls (0.27 ± 0.13 and 0.07 ± 1.02%, respectively) (Figure 7). A decrease in Bcl-2 expression with a concurrent decrease in phosphorylation was observed in presensitized A549- (1.58 ± 0.17%) and MCF-7 (3.7 ± 0.29%) cells when compared to A549 (0.67 ± 0.25%)- and MCF-7 (0.97 ± 0.61%) cells exposed to DMSO. Compared to the individual treatment conditions, presensitized A549- and MCF-7 cells demonstrated an increased number of cells with a decrease in Bcl-2 expression and -phosphorylation. In the MCF-7 cells, more cells displayed a decrease in phosphorylation in the combination treatment when compared to cells exposed to ESE-15-ol (0.49 ± 0.32%) and 6 Gy radiation (0.4 ± 0.15%) alone (Figure 7).

## 3. Discussion

Results obtained from this in vitro study indicated that long-term survival of cells exposed to 6 Gy radiation decreased with pre-exposure to a low-dose estrone analog in both the breast- and lung cancer cells, although to a lesser extent in the latter. Chronological investigations revealed an early increase in ROS and a decreased Bcl-2 expression and phosphorylation, implicating intrinsic apoptotic pathway signaling as one of the potential pathways involved in the cell fate decision. These pathways were augmented by the addition of the drug prior to radiation, as was seen in the comparison to the individual treatment conditions.

Several 2ME2 analogs were designed in silico in our laboratory to overcome the pharmacokinetic constraints of 2ME2, improve its oral bioavailability and enhance its cytotoxicity [19,28]. These molecules were designed to bind to the colchicine site with greater affinity than 2ME2. Additionally, the design aimed at bypassing the first-pass metabolism by CAII binding within the erythrocytes. Preferential binding to CAIX would ensure localization of the drug to the acidic micromilieu of solid tumors, thereby providing more of a targeted anticancer effect. These analogs have displayed cytotoxicity at nanomolar concentrations in multiple neoplastic cell lines, including a multiple dug resistant uterine carcinoma cell line [9]. The cytotoxic effect of ESE-15-ol was confirmed in the present study revealing a GI_50_ of 0.333 µM and 0.111 µM for A549- and MCF-7 cells, respectively.

GI_50_ doses of these analogs disrupted microtubule morphology and spindle formation in treated HeLa- [9], MDA-MB-231- [19], and SNO [29] cell lines. This resulted in the accumulation of cells at G_2_/M within the cell cycle due to the spindle-assembly checkpoint (SAC) not being satisfied [28]. Furthermore, very low doses of the analogs (not sufficient to cause cell death) decrease microtubule dynamics, thereby potentially affecting the associated trafficking of organelles, autophagosomes, and other signaling molecules [24,30]. Exposure to these compounds induces apoptosis via both the intrinsic and extrinsic pathways (including the Bcl-2 family proteins in the former), increased ROS formation, and autophagic cell death [16].

Oxidative stress damages proteins, nucleic acids, and lipids, which leads to loss of membrane integrity, chromosomal instability, and mutations [31]. Treatment of neuroblastoma cells (SK-N-SH and SH-SY5Y cell lines) in vitro with 2ME2 resulted in significantly increased generation of reactive oxygen species (ROS) [32]. 2ME2 treatment was associated with a decreased mitochondrial membrane potential (in a dose-dependent manner), suggesting that ROS-induced mitochondrial injury mediated apoptosis induced by 2ME2 [32]. High levels of cytosolic ROS were detected after radiosensitive PC3 cells were exposed to 4- and 8 Gy radiation [33]. PC3- and Du145 cells revealed maximum intracellular ROS production 15 min after radiation, after which levels declined over time [33]. Results obtained from the current study revealed increased ROS levels in all treated controls. Presensitized A549 cells displayed a statistically significant increase in ROS production over the 48-h time period when compared to the individual treatment conditions. Intracellular ROS production was significantly increased in MCF-7 cells 24-h after radiation, after which a decline was observed. These results demonstrate a temporal response of the cells to the treatment, which differed between the cell lines investigated. A549 cells show a delayed and attenuated response when compared to the MCF-7 cells.

The Bcl-2 family consists of proapoptotic and antiapoptotic proteins [34]. Bcl-2 is an antiapoptotic protein. Bcl-2 binds to various proapoptotic family members (e.g., Bax) and inhibits their insertion into the mitochondrial membrane [35]. Upon receiving an apoptotic signal, Bax is released by Bcl-2, allowing the former to form a complex on the mitochondrial membrane. Cytochrome *c* is consequently released into the cytoplasm, triggering caspase activity and cell death [35]. 2ME2 treatment inhibits Bcl-2 expression, while increasing Bax levels in human neuroblastoma cells [32]. This decreases the Bcl-2/Bax ratio causing permeabilization of the mitochondrial membrane and activation of caspases 9 and -3 resulting in apoptotic cell death [32]. Hara et al. demonstrated that overexpression of Bcl-2 in HeLa cells renders them more resistant to radiation compared to wild type HeLa cells [36]. By using Bcl-2 inhibitors such as Tetrocarcin A (TC-A) the team was able to increase the sensitivity of Bcl-2-overexpressing HeLa cells to radiation [36]. The present study revealed no effect on Bcl-2 expression or -phosphorylation at serine 70 in A549- and MCF-7 cells exposed to the individual treatment conditions. However, Bcl-2 expression and -phosphorylation were significantly decreased in A549- and MCF-7 cells presensitized to radiation with low-dose ESE-15-ol. These results suggest that ESE-15-ol in combination with 6 Gy radiation produce a stronger apoptotic signal by decreasing Bcl-2 function when compared to the individual treatment conditions. 

Microtubule disrupting agents hold promise as radiosensitizing agents using the rationale that the G_2_/M phase is the most radiosensitive phase of the cell cycle [15,16]. Cazares et al. demonstrated the synergistic effect of 2ME2 and ionizing radiation on cell death using in vitro and in vivo prostate cancer models [17]. However, at doses insufficient to cause cell death, inhibition of the dynamic treadmilling of the microtubules may affect the subcellular localization of proteins involved in DNA repair [37]. Spindle abrogation in response to nocodazole induced a G_1_-phase block represented by an increased in phosphorylated retinoblastoma protein (pRb) and p53 signaling [38]. This provides evidence that these agents may affect many homeostatic mechanisms within interphase cells and induce apoptosis in alternative pathways to the metaphase block. Future studies will investigate this hypothesis by quantifying radiation-induced DNA damage and investigating the subcellular localization of the repair proteins.

In this study, cell cycle analysis revealed a significant increase in the sub-G_1_ phase of presensitized MCF-7 cells following radiation compared to the individual treatment conditions. These findings were not translated to the A549 cells. This was mirrored by the increased quantification of externalized phosphatidylserine indicating apoptosis induction. This method also demonstrated a significant decrease in cell viability in presensitized MCF-7 cells. In A549 cells, all treated samples displayed a significant but small reduction in cell viability and increase in apoptotic cells compared to the negative control, but did not significantly differ from each other. The slightly more significant increase in the sub-G_1_ population on cell cycle analysis may not only indicate apoptotic cells, but also represents cells with increased DNA fragmentation and micronuclei formation [25]. Only the radiation-treated samples showed an increased G_2_/M phase in this cell line. Therefore, the potential induction of a G_1_ block exists, or a temporal progression including a mitotic block not represented in the measured time-lines. 

The ESE-15-ol-treated controls demonstrated that a very low dose of the compound was used, as well as the reversible intracellular effect of the drug [24,30]. As the drug is washed off after 24-h, the cells were able to recover to some extent over the following 48-h as the microtubules restored their structure and function. This is clearly evident in the morphological studies in which drug-exposed cells recovered to similar presentations as the negative control after 48-h.

Apoptosis induction by the different treatment conditions was visualized by light- and transmission electron microscopy. Apoptotic characteristics such as apoptotic body formation, increased cellular protrusions and hypercondensed chromatin were evident in all treated samples. Presensitized A549- and MCF-7 cells decreased in cellular density and apoptotic body formation.

Clonogenic studies represent the long-term fate of the cells following treatment, with colony formation indicating the ability of the cells to survive, restore and proliferate. 2ME2 decreased colony formation significantly in human colon carcinoma (HCT116 and SW613-B3) cells [7]. Cazarez et al. demonstrated increased sensitivity to radiation of PC3 human prostate carcinoma cells with abolished MAPK activity with a significant decrease in colony formation post-radiation [17]. The same effect on colony formation was observed in PC3 cells treated with 2ME2 prior to radiation, illustrating 2ME2’s ability to sensitize cells to radiation [17]. These findings seem to concur with the findings demonstrated in this study. Colony formation was significantly decreased in A549- and MCF-7 cells exposed to low-dose ESE-15-ol and 6 Gy radiation. The effectivity of ESE-15-ol exposure to cells prior to radiation is evident by the decreased colony formation, which is greater than the individual treatment conditions.

Results of this study indicate that ESE-15-ol may indeed be a useful agent with which to radiosensitize tumor cells. Induction of apoptosis occurs in a time-dependent sequence, with increased ROS production and decreased Bcl-2 signaling being more prominent in the combination cells at the earlier time frame. Although apoptosis is not clearly enhanced in A549 cells at 48-h post radiation, this treatment results in a long-term decrease in cell viability and attenuated proliferation. The low dose required for augmenting the signaling cascade, increasing ROS, and decreasing colony formation, along with its reversibility, makes the drug ideal for minimizing treatment side-effects and regulating dosage responses. Potentially, both drug and radiation may be administered at lower doses for a potential augmented response with fewer unwanted effects.

## 4. Materials and Methods

### 4.1. Cell Lines, Culture Methods and Chemicals

The commercially available human estrogen receptor-positive MCF-7 epithelial breast cancer cell line [39] and the A549 lung adenocarcinoma epithelial cell line [40] were used in this in vitro study. Both cell lines were acquired from Cellonex (Johannesburg, South Africa).

MCF-7- and A549 cells were cultured in Gibco^®^ Dulbecco’s Modified Eagle Medium (DMEM) (Thermo Fisher Scientific, Waltham, MA, USA) supplemented with 10% heat-inactive fetal bovine serum (FBS) (Hyclone, Thermo Fisher Scientific, Waltham, MA, USA), streptomycin (100 µg/mL), penicillin G (100 U/mL), and fungizone (250 µg/L) (Sigma-Aldrich, St. Louis, MO, USA). Cells were incubated in a forma Scientific water-jacketed incubator (5% CO_2_, 37 °C) (Waltham, MA, USA). Phosphate buffered saline (PBS) was diluted from a 10× stock solution (80 g/L NaCl, 2 g/L KCl, 2 g/L KH2PO4, and 11.5 g/L Na2HPO4) to a 1× working solution prior to use. Cells were trypsinised with trypsin-ethylenediaminetetraacetic acid (EDTA) (Gibco^®^, Thermo Fisher Scientific, MA, USA) for cell detachment. All other chemicals not specifically mentioned were of analytical grade and were purchased from Sigma-Aldrich (St. Louis, MO, USA).

The noncommercially available in silico-designed 2ME2 analog (ESE-15-ol) was synthesized by Ithemba (PTY) Ltd. Pharmaceuticals (Gauteng, South Africa). ESE-15-ol was dissolved in dimethyl sulfoxide (DMSO) to a 10 mM stock solution and stored at −20 °C. MCF-7- and A549 cells were seeded at a density of 5000 cells/80 µL for spectrophotometric analysis, 300,000 cells/3 mL for light microscopy, and 700,000/5 mL for electron microscopy and flow cytometric analyses. The lowest compound dose that decreased cell viability significantly and the lowest radiation dose that decreased cell viability to 80% was determined by a dose curve. The experimental setup comprised of a 24-h cell attachment policy [24], followed by a 24-h exposure to 0.081 µM, and 0.056 µM of ESE-15-ol for A549- and MCF-7 cells, respectively. Cells were rinsed with PBS and DMEM was replaced prior to radiation. The treatment controls comprised of ESE-15-ol exposure for 24-h, before washing the drug off and incubating the cells for 48-h in parallel with the combination treatment. The radiation only control comprised of addition of DMSO when the drug was administered, and undergoing the same experimental timeline.

Samples were irradiated using a Siemens Oncor Impression Linear Accelerator with a field size of 10 cm × 10 cm. The gantry angle was set to 180° and the source-to-surface distance was 100 cm. Samples were irradiated to 6 Gy and 1.5 cm of tissue equivalent bolus was used to ensure dose homogeneity. Experiments were terminated 48-h after radiation as apoptotic cell death was visible at this time.

### 4.2. Controls

MCF-7- and A549 cells propagated in DMEM served as a negative control whereas DMSO (0.05% *v*/*v*) served as a vehicle control. No significant differences were observed between the vehicle control and negative control during all experimental setups. Actinomycin D served as a positive control for apoptosis. 

### 4.3. Cytotoxicity Study

Exponentially growing MCF-7- and A549 cells were exposed to an ESE-15-ol dilution series (10-, 5-, 2.5-, 1.25-, 0.625-, 0.313-, 0.156-, 0.078-, 0.039-, and 0.02 µM) for 24-h and 48-h in 96-well microtiter plates (TPP Techno Plastic Products AG, Trasadingen, Switzerland). DMSO (0.05% *v*/*v*) was used as a negative control (100% cell viability) and 1% SDS as a positive control (complete cell death). Cells were washed with PBS and incubated with 0.5 g/mL 3-(4,5-dimethylthiazol-2-yl)-2,5-diphenyltetrazolium bromide (MTT) in white Roswell Park Memorial Institute medium (RPMI) (Gibco^®^, Thermo Fisher Scientific, MA, USA) for 4-h at 37 °C. Formazan crystals were dissolved with MTT solubilization solution (1800 mL isopropanol, 16 mL 37% HCl and 200 mL triton X-100). The absorbance was read at 570 nm using an ELx800 Universal Microplate Reader (Bio-Tek Instruments Inc., Winooski, VT, USA). Three biological repeats were performed, each with *n* = 6. The concentration of drug which caused 50% growth inhibition over 24-h or 48-h was calculated in both cell lines (GI_50_).

### 4.4. Apoptosis Studies: Annexin V—Fluorescein Isothiocyanate (FITC)

The FITC Annexin V apoptosis detection kit (BioLegend, San Diego, CA, USA) was used for apoptosis detection. Treated MCF-7- and A549 cells were resuspended in Annexin V binding buffer and exposed to Annexin V-FITC antibody (5 µL) and propidium iodide (PI) solution (10 µL) for 15 min at room temperature protected from light. Annexin V binding buffer (400 µL) was added to each tube. Advanced apoptotic and necrotic cells (FL3 fluorescence) and apoptotic cells (FL1 fluorescence) were quantified using a FC500 system flow cytometer (Beckman Coulter, Brea, CA, USA).

### 4.5. Cell Cycle Progression

Trypsinized cells were washed with ice-cold PBS containing 0.1% FBS. Cells were fixed with ice-cold 70% ethanol for 24-h at 4 °C. Cells were washed and resuspended in PBS containing PI (40 µg/mL), triton X-100 (0.1%), and RNase A (100 µg/mL) and incubated for 40 min at 37 °C. PI fluorescence (FL3) was measured on a FC500 system flow cytometer (Beckman Coulter, Brea, CA, USA).

### 4.6. Polarization-Optical Transmitted Light Differential Interference Contrast Microscopy (PlasDIC)

Polarization-optical transmitted light differential interference contrast micrographs (40× magnification) were obtained by using a Zeiss Axiovert-40 microscope (Zeiss, Oberkochen, Germany). Micrographs were taken 6-h, 24-h, and 48-h post radiation.

### 4.7. Transmission Electron Microscopy (TEM)

MCF-7- and A549 cells were fixed in 2.5% glutaraldehyde in 0.075 M phosphate buffer (pH 4.4) for 1-h at room temperature. Cells were rinsed three times in 0.075 M phosphate buffer. Cells were fixed in 0.5% aqueous osmium tetroxide for 1-h and washed with distilled water (3 times) thereafter. Fixed cells were dehydrated with increasing ethanol concentrations (30%, 50%, 10%, 90%, 100%, 100%, and 100%) for 10 min at each concentration. Cells were infiltrated with 30% EMBED 812 in ethanol for 1-h, followed by 60% EMBED 812 in ethanol for 1-h, and 100% EMBED 812 for 4-h. Specimens were embedded at 60 °C for 39-h. A microtome was used to prepare ultrathin sections which were mounted on a copper grid. Samples were contrasted with 4% aqueous uranyl acetate (10 min) and Reynold’s lead citrate (2 min) separated by rinsing steps. A JEM-2100F field emission transmission electron microscope (JEOL Ltd, Tokyo, Japan) was used to view the samples. 

### 4.8. Clonogenic Studies

MCF-7- and A549 cells were seeded at different densities depending on treatment. Negative and vehicle controls were seeded at a density of 100 cells/3 mL of DMEM. Cells exposed to ESE-15-ol were seeded at a density of 400 cells/3 mL while the positive control, radiation control, and cells exposed to ESE-15-ol 24-h prior to radiation were seeded at a density of 1000 cells/3 mL. Cells were allowed to form colonies for 10 days after radiation. Colonies were fixed with 1% gluteraldehyde (100 µL) for 15 min. After the gluteraldehyde was discarded, 100 µL 0.1% crystal violet was added to the cells (30 min). The plates were immersed under running tap water. Colony formation was quantified based upon the area covered by the colonies in a 6 well plate, as well as the intensity of colonies (how densely populated the colonies are) using the ColonyArea plug-in for Image J 1.48v (National Institute of Health, Bethesda, MD, USA). The area and intensity percentages obtained from the samples were divided by the initial amount of cells seeded in order to normalize the calculations.

### 4.9. Superoxide Detection

Termination of the experiment followed 6-h, 24-h, and 48-h after radiation. MCF-7- and A549 cells were resuspended in 1 mL PBS. Hydroethidine stock solution (1 µL) was added to cells and incubated for 15 min at 37 °C. Cells were analyzed using a FC500 System flow cytometer (Beckman Coulter).

### 4.10. B Cell Lymphoma 2 (Bcl-2) Activation

The FlowCellect Bcl-2 Activation dual detection kit (Merck Millipore, Burlington, MA, USA) was used to determine Bcl-2 expression and -phosphorylation at serine 70. MCF-7- and A549 cells were washed with the supplied wash buffer and resuspended in fixation buffer for 20 min. Fixed cells were washed in 1× assay buffer. Ice-cold permeabilization buffer (0.5 mL) was added to the pellet for 10 min. Assay buffer (100 µL) containing anti-Bcl-2 antibody conjugated to AlexaFluor^®^ 488 and an anti-pBcl-2 (ser70) conjugated to phycoerythrin were added to the cells for 60 min at 4 °C (protected from light). Cells were washed twice with assay buffer. Fluorescence at FL1 (Bcl-2 antibody) and FL3 (pBcl-2, Ser 70) were measured with a FC500 System flow cytometer (Beckman Coulter).

### 4.11. Statistical Analysis

Qualitative data were obtained from morphological studies including TEM and PlasDIC, each performed in duplicate. Quantitative data (time-dependent studies, cytotoxic studies, and flow cytometry) were obtained from a minimum of three independent biological replicates. Flow cytometric data obtained from at least 10,000 cells were analyzed using Kaluza Analysis Flow Cytometric Software, version 1.3 (CA, USA). Data were statistically analyzed using the analysis of variance (ANOVA)-single factor model for significance, followed by a two-tailed Student’s *t*-test. *p*-values < 0.05 were regarded as statistically significant. 

### 4.12. Ethics Approval

Ethics approval was obtained from The Research Ethics committee, Faculty of Health Sciences, University of Pretoria, Pretoria, South Africa (Ethics reference number: 265/2014, approved on 30/07/2014). 

## 5. Conclusions

This in vitro study revealed that the combined effect of low-dose ESE-15-ol and radiation are more pronounced than the individual treatment modalities. ROS generation responded in a temporal manner in both cell lines, while Bcl-2 expression and -phosphorylation at serine 70 decreased. However, the onset of apoptosis as detected by the phosphatidyl serine flip and sub-G_1_ accumulation occurred more rapidly in the MCF-7 cells. Importantly, long-term survival and regeneration as assessed by clonogenic studies indicated a significant response to the combination therapy, with decreased colony formation in both cell lines. Thus the hypothesis was formed that pre-exposure to low-dose ESE-15-ol in part enhances the intrinsic apoptotic pathway via ROS and Bcl-2 signaling in a time-dependent sequence, resulting in long-term decreased cell survival and regeneration. However, additional mechanisms through which ESE-15-ol may sensitize cells to radiation remain undefined. Contribution of the induction of cellular senescence, as well as effects of the functional disruption of microtubule dynamics by ESE-15-ol on DNA repair mechanisms and protein shuttling will be examined.

## Figures and Tables

**Table 1:** Dose response curves of A549- and MCF-7 cells exposed to ESE-15-ol for 24- and 48-h. No statistical significant differences were calculated between GI_50_ values determined at different exposure times.

Cell Line		A549	MCF-7
**Graphical Representation**		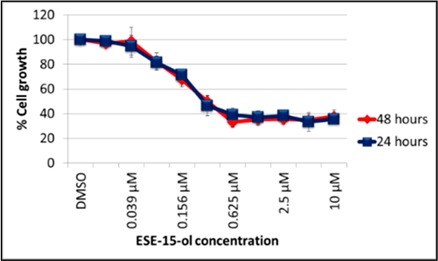	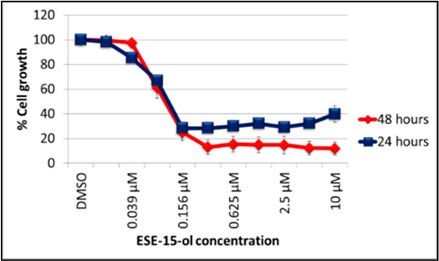
**Exposure Time**	**24-h Exposure**	0.333 µM	0.111 µM
	**48-h Exposure**	0.318 µM	0.101 µM

**Figure A1:**
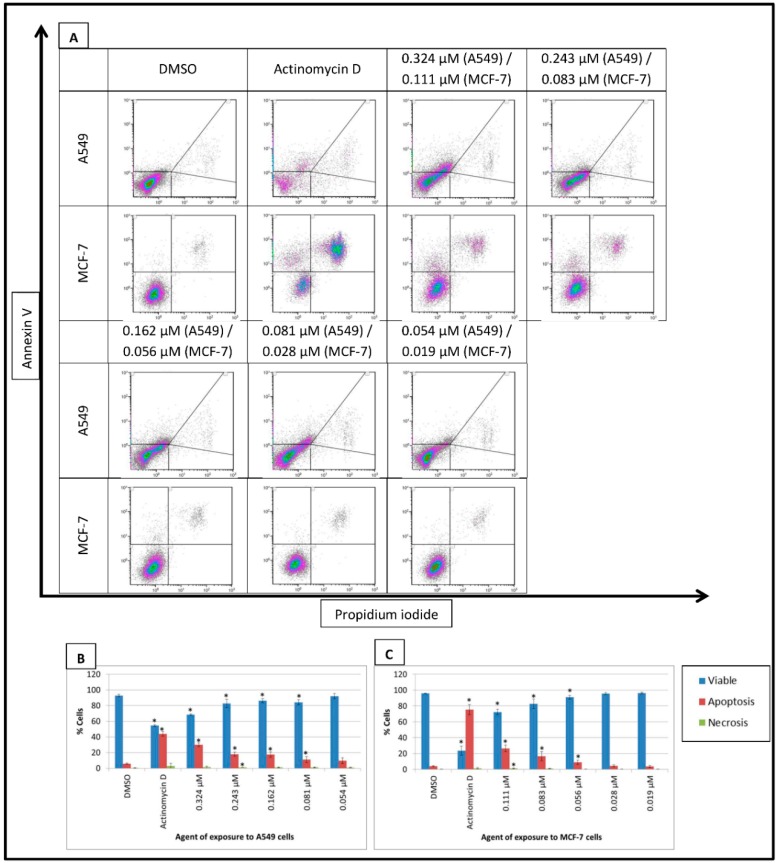
Annexin V-FITC flow cytometric density dot plots of A549- and MCF-7 cells exposed to an ESE-15-ol dose range. Annexin V-FITC (FL1) measured apoptotic cells and was plotted against propidium iodide (FL3) that measured necrotic cells (**A**). DMSO served as a vehicle control and actinomycin D as a positive control for apoptosis. The lowest concentration of ESE-15-ol that decreased cell viability significantly was determined to be 0.081 µM for A549 cells and 0.056 µM for MCF-7 cells. Graphical representations of A549 dot plots (**B**) and MCF-7 dot plots (**C**). Bars indicate average of three biological repeats, with standard deviation represented by T-bars. Statistically significant differences are indicated by * (*p*-value < 0.05).

**Figure A2:**
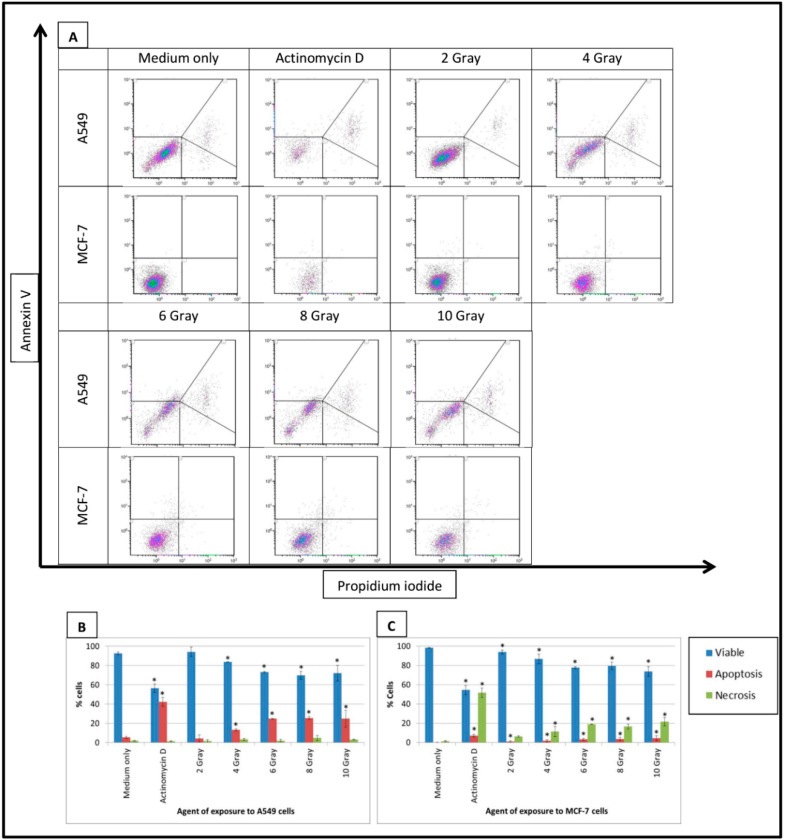
Annexin V-FITC flow cytometric density dot plots of A549- and MCF-7 cells exposed to a radiation dose range. Annexin V-FITC (FL1) measured apoptotic cells and was plotted against propidium iodide (FL3) that measured necrotic cells (**A**). Cells propagated in medium only served as a negative control and actinomycin D as a positive control for apoptosis. The lowest gray that decreased cell viability to 80% or less was determined at 6 gray for A549- and MCF-7 cells. Graphical representations of A549 dot plots (**B**) and MCF-7 dot plots (**C**). Bars indicate average of three biological repeats with standard deviation represented by T-bars. Statistically significant differences are indicated by * (*p*-value < 0.05).

## Abbreviations

2ME2	2-Methoxyestradiol
ANOVA	Analysis of variance
BCA	Bicinchoninic acid
Bcl-2	B Cell lymphoma 2
BSA	Bovine serum albumin
CA	Carbonic anhydrase
Cdc2	Cell division cycle 2
DMEM	Dulbecco’s Modified Eagle Medium
DMSO	Dimethyl sulfoxide
DNA	Deoxyribonucleic acid
EDTA	Ethylenediaminetetraacetic acid
ESE-15-ol	2-Ethyl-3-*O*-sulfamoyl-estra-1,3,5(10),15-tetraen-17-ol
FBS	Fetal bovine serum
FITC	Fluorescein isothiocyanate
GADD45	Growth arrest and DNA damage-inducible 45
GI_50_	Half-maximal inhibitory growth concentrations
kg	Kilogram
MAPK	Mitogen-activated protein kinase
mdm2	Murine double minute 2
MEK	Mitogen-activated protein kinase kinase
mg	Milligram
min	Minute
MTT	3-(4,5-dimethylthiazol-2-yl)-2,5-diphenyltetrazolium bromide
PBS	Phosphate buffered saline
PgP	P-glycoprotein
PI	Propidium iodide
PlasDIC	Polarization-optical transmitted light differential interference contrast microscopy
ROS	Reactive oxygen species
RPMI	Roswell Park Memorial Institute medium
SAC	Spindle-assembly checkpoint
TC-A	Tetrocarcin A
TEM	Transmission electron microscopy

## Appendix A

### ESE-15-ol Dose Response Curve

The lowest concentration of ESE-15-ol that decreased cell viability significantly was used to presensitized A549- and MCF-7 cells to radiation. A549- and MCF-7 cells were exposed to an ESE-15-ol dose curve (GI_50_, ¾ GI_50_, ½ GI_50_, ¼ GI_50_, ⅙ GI_50_) in a 24-h exposure time frame.

The lowest concentration of ESE-15-ol that decreased A549 cell viability significantly was calculated at 0.081 µM (84.29 ± 3.28%). Apoptosis was significantly increased in A549 cells exposed to 0.081 µM (11.26 ± 3.75%) when compared to DMSO (5.93 ± 0.8%) (Figure A1). MCF-7 cell viability was significantly decreased when exposed to 0.056 µM ESE-15-ol (90.88 ± 2.46%) (Figure A1). Apoptosis was also significantly increased after MCF-7 cells were exposed to 0.056 µM ESE-15-ol (8.8 ± 2.59%).

## Appendix B

### Radiation Dose Response Curve

The lowest gray (Gy) that decreased cell viability to 80% or less was used to radiate A549- and MCF-7 cells. A549- and MCF-7 cells were exposed to a radiation dose curve (2 Gy, 4 Gy, 6 Gy, 8 Gy, 10 Gy) and terminated 48-h after treatment.

The lowest Gy that decreased cell viability to 80% or less when compared to the negative control was determined at 6 Gy for A549- (73.32 ± 0.7%) and MCF-7 (77.76 ± 1.22%) cells (Figure A2). A549- and MCF-7 cells exposed to 6 Gy radiation revealed an increase in apoptosis (24.69 ± 0.4% and 3.29 ± 1.1%, respectively).

## References

[1] McGinn C.J., Lawrence T.S. (2001). Recent advances in the use of radiosensitizing nucleosides. Semin. Radiat. Oncol..

[2] Lawrence T.S., Blackstock A.W., McGinn C. (2003). The mechanism of action of radiosensitization of conventional chemotherapeutic agents. Semin. Radiat. Oncol..

[3] Liebmann J., Cook J.A., Fisher J., Teague D., Mitchell J.B. (1994). In vitro studies of Taxol as a radiation sensitizer in human tumor cells. J. Natl. Cancer Inst..

[4] Chen Y., Pandya K., Keng P.C., Johnstone D., Li J., Lee Y.J., Smudzin T., Okunieff P. (2003). Phase I/II clinical study of pulsed paclitaxel radiosensitization for thoracic malignancy: A therapeutic approach on the basis of preclinical research of human cancer cell lines. Clin. Cancer Res..

[5] Stromberg J.S., Lee Y.J., Armour E.P., Martinez A.A., Corry P.M. (1995). Lack of radiosensitization after paclitaxel treatment of three human carcinoma cell lines. Cancer.

[6] Greco F.A., Stroup S.L., Gray J.R., Hainsworth J.D. (1996). Paclitaxel in combination chemotherapy with radiotherapy in patients with unresectable stage III non-small-cell lung cancer. J. Clin. Oncol..

[7] Parks M., Tillhon M., Dona F., Prosperi E., Scovassi A.I. (2011). 2-methoxyestradiol: New perspectives in colon carcinoma treatment. Mol. Cell. Endocrinol..

[8] Cushman M., He H.M., Katzenellenbogen J.A., Lin C.M., Hamel E. (1995). Synthesis, antitubulin and antimitotic activity, and cytotoxicity of analogs of 2-methoxyestradiol, an endogenous mammalian metabolite of estradiol that inhibits tubulin polymerization by binding to the colchicine Binding Site. J. Med. Chem..

[9] Theron A., Prudent R., Nolte E., van den Bout I., Punchoo R., Marais S., du Toit P., Hlophe Y., van Papendorp D., Lafanchere L. (2015). Novel in silico-designed estradiol analogues are cytotoxic to a multidrug-resistant cell line at nanomolar concentrations. Cancer Chemother. Pharmacol..

[10] Chauhan D., Catley L., Hideshima T., Li G., Leblanc R., Gupta D., Sattler M., Richardson P., Schlossman R.L., Podar K. (2002). 2-methoxyestradiol overcomes drug resistance in multiple myeloma cells. Blood.

[11] Chiche J., Ilc K., Laferriere J., Trottier E., Dayan F., Mazure N.M., Brahimi-Horn M.C., Pouysségur J. (2009). Hypoxia-inducible carbonic anhydrase IX and XII promote tumor cell growth by counteracting acidosis through the regulation of the intracellular pH. Cancer Res..

[12] Stander B.A., Marais S., Vorster C.J., Joubert A.M. (2010). In vitro effects of 2-methoxyestradiol on morphology, cell cycle progression, cell death and gene expression changes in the tumorigenic MCF-7 breast epithelial cell line. J. Steroid Biochem. Mol. Biol..

[13] Dunn J.F., Merriam G.R., Eil C., Kono S., Loriaux D.L., Nisula B.C. (1980). Testosterone-estradiol binding globulin binds to 2-methoxyestradiol with greater affinity than to testosterone. J. Clin. Endocrinol. Metab..

[14] Leese M.P., Leblond B., Smith A., Newman S.P., Di Fiore A., De Simone G., Supuran C.T., Purohit A., Reed M.J., Potter B.V.L. (2006). 2-substituted estradiol bis-sulfamates, multitargeted antitumor agents: Synthesis, in vitro SAR, protein crystallography, and in vivo activity. J. Med. Chem..

[15] Jin C., Wu H., Liu J., Bai L., Guo G. (2007). The effect of paclitaxel-loaded nanoparticles with radiation on hypoxic MCF-7 cells. J. Clin. Pharm. Ther..

[16] Masunaga S., Ono K., Suzuki M., Nishimura Y., Kinashi Y., Takagaki M., Hori H., Nagasawa H., Uto Y., Tsuchiya I. (2001). Radiosensitization effect by combination with paclitaxel in vivo, including the effect on intratumor quiescent cells. Int. J. Radiat. Oncol. Biol. Phys..

[17] Casarez E.V., Dunlap-Brown M.E., Conaway M.R., Amorino G.P. (2007). Radiosensitization and modulation of p44/42 mitogen-activated protein kinase by 2-Methoxyestradiol in prostate cancer models. Cancer Res..

[18] Dent P., Reardon D.B., Park J.S., Bowers G., Logsdon C., Valerie K., Schmidt-Ullrich R. (1999). Radiation-induced release of transforming growth factor alpha activates the epidermal growth factor receptor and mitogen-activated protein kinase pathway in carcinoma cells, leading to increased proliferation and protection from radiation-induced cell death. Mol. Biol. Cell.

[19] Stander A., Joubert F., Joubert A. (2011). Docking, synthesis, and in vitro evaluation of antimitotic estrone analogs. Chem. Biol. Drug Des..

[20] Stander B.A., Joubert F., Tu C., Sippel K.H., McKenna R., Joubert A.M. (2013). Signaling pathways of ESE-16, an antimitotic and anticarbonic anhydrase estradiol analog, in breast cancer cells. PLoS ONE.

[21] Pastorekova S., Ratcliffe P.J., Pastorek J. (2008). Molecular mechanisms of carbonic anhydrase IX-mediated pH regulation under hypoxia. BJU Int..

[22] Ho Y.T., Purohit A., Vicker N., Newman S.P., Robinson J.J., Leese M.P., Ganeshapillai D., Woo L.W.L., Potter B.V.L., Reed M.J. (2003). Inhibition of carbonic anhydrase II by steroidal and non-steroidal sulphamates. Biochem. Biophys. Res. Commun..

[23] Elger W., Barth A., Hedden A., Reddersen G., Ritter P., Schneider B., Zuchner J., Krahl E., Muller K., Oettrl M. (2001). Estrogen sulfamates: A new approach to oral estrogen therapy. Reprod. Fertil. Dev..

[24] Theron A.E., Nolte E.M., Lafanechere L., Joubert A.M. (2013). Molecular crosstalk between apoptosis and autophagy induced by a novel 2-methoxyestradiol analogue in cervical adenocarcinoma cells. Cancer Cell Int..

[25] Riccardi C., Nicoletti I. (2006). Analysis of apoptosis by propidium iodide staining and flow cytometry. Nat. Protoc..

[26] Xu Z., Yan Y., Xiao L., Dai S., Zeng S., Qian L., Wang L., Yang X., Gong Z. (2017). Radiosensitizing effect of diosmetin on radioresistant lung cancer cells via Akt signaling pathway. PLoS ONE.

[27] Antognelli C., Gambelunghe A., Talesa V.N., Muzi G. (2014). Reactive oxygen species induce apoptosis in bronchial epithelial BEAS-2B cells by inhibiting the antiglycation glyoxalase I defence: Involvement of superoxide anion, hydrogen peroxide and NF-κB. Apoptosis.

[28] Stander B., Joubert F., Tu C., Sippel K., McKenna R., Joubert A. (2012). In vitro evaluation of ESE-15-ol, an estradiol analogue with nanomolar antimitotic and carbonic anhydrase inhibitory activity. PLoS ONE.

[29] Wolmarans E., Sippel K., McKenna R., Joubert A. (2014). Induction of the intrinsic apoptotic pathway via a new antimitotic agent in an esophageal carcinoma cell line. Cell Biosci..

[30] Theron A.E. (2016). In vitro and In vivo Anti-Cancer Properties of Novel Spindle Disruptors. Ph.D. Thesis.

[31] Pan J., Hong M., Ren J. (2009). Reactive oxygen species: A double-edged sword in oncogenesis. World J. Gastroenterol..

[32] Zhang Q., Ma Y., Cheng Y., Li W., Zhang Z., Chen S. (2011). Involvement of reactive oxygen species in 2-methoxyestradiol-induced apoptosis in human neuroblastoma cells. Cancer Lett..

[33] Jayakumar S., Kunwar A., Sandur S.K., Pandey B.N., Chaubey R.C. (2014). Differential response of DU145 and PC3 prostate cancer cells to ionizing radiation: Role of reactive oxygen species, GSH and Nrf2 in radiosensitivity. Biochim. Biophys. Acta (BBA) Gen. Subj..

[34] Huang D.C., Strasser A. (2000). BH3-Only proteins-essential initiators of apoptotic cell death. Cell.

[35] Gross A., Jockel J., Wei M.C., Korsmeyer S.J. (1998). Enforced dimerization of BAX results in its translocation, mitochondrial dysfunction and apoptosis. EMBO J..

[36] Hara T., Omura-Minamisawa M., Chao C., Nakagami Y., Ito M., Inoue T. (2005). Bcl-2 inhibitors potentiate the cytotoxic effects of radiation in Bcl-2 overexpressing radioresistant tumor cells. Int. J. Radiat. Oncol. Biol. Phys..

[37] Brooks W.S., Banerjee S., Crawford D.F. (2007). G2E3 is a nucleo-cytoplasmic shuttling protein with DNA damage responsive localization. Exp. Cell Res..

[38] Farhat M., Poissonnier A., Hamze A., Ouk-Martin C., Brion J., Alami M., Feuillard J., Jayat-Vignoles C. (2014). Reversion of apoptotic resistance of TP53-mutated Burkitt lymphoma B-cells to spindle poisons by exogenous activation of JNK and p38 MAP kinases. Cell Death Dis..

[39] MCF7 ATCC ® HTB-22™ Homo Sapiens Mammary Gland/Breast; Deriv. http://www.atcc.org/products/all/HTB-22.aspx.

[40] A549 ATCC ® CCL-185™ Homo Sapiens Lung Carcinoma. http://www.atcc.org/products/all/CCL-185.aspx#generalinformation.

